# Technological Risks (GMO, Gene Editing), What Is the Problem With Europe? A Broader Historical Perspective

**DOI:** 10.3389/fbioe.2020.557115

**Published:** 2020-11-09

**Authors:** Marcel Kuntz

**Affiliations:** Laboratoire de Physiologie Cellulaire et Végétale, Université Grenoble Alpes, CNRS, CEA, INRAE, Grenoble, France

**Keywords:** enlightenment, judiciary, political actions, postmodernism, regulation, technology

## Abstract

Europe is often the center of origin of restrictions regarding technologies (e.g., biotechnologies: GMOs and, more recently, gene editing). The causes have already been analyzed in relation to European regulations, but not to its deeply embedded roots. This is what the present article attempts to do. It first depicts the broader historical background in Europe, the rise of a new ideology aiming to avoid repetition of the tragedies of the past, and the way these postmodern ideas have been transposed to science, with a focus on the issue of technological risk. In contrast to Europe, the United States has not enacted biotechnology-inhibiting laws, and the reasons for such a difference are discussed.

## Introduction

To understand why Europe is restricting the use of some technologies, while the United States are not following the same path for the same technologies, plant biotechnology is a useful example. Obviously, Europe is center of origin of the GMO backlash. A short-term reason can be sought in its Directive from 1990, which has created a judicial object called a “genetically modified organism.” It was replaced by a new Directive in 2001 but kept its meaningless definition of a GMO (Tagliabue, 2016a). This regulatory approach focuses on an “organism in which the genetic material has been altered in a way that does not occur naturally,” giving the impression that GMOs are intrinsically different and risky, and consequently created the possibility of rejection of transgenesis, a promising technology, by distrustful consumers in the wake of the “mad cow” crisis. The July 2018 judgment of the Court of Justice of the European Union (CJEU) (“Organisms obtained by mutagenesis are GMOs and are, in principle, subject to the obligations laid down by the GMO Directive”^1^) was a new blow for biotechnologists. However, the question that emerges is: Why did all these events happen in Europe? To understand we need to characterize the ideological context, and to do so to look at a broader historical perspective.

### A Brief History of Europe During the XX Century

During the last century, Europe suffered from two devastating World Wars, the mass crimes of two totalitarian states and the inhumane nature of their concentration camps, and several genocides. In a legitimate attempt to avoid repetition of such tragic events, European integration was postulated. Even Sir Winston Churchill advocated “a kind of United States of Europe” in 1946 [“If Europe were once united in the sharing of its common inheritance, there would be no limit to the happiness, to the prosperity and the glory which its three or four hundred million people would enjoy” (Churchill, 1946)]. The Council of Europe was founded in 1949 and the European Economic Community in 1957. European construction has indeed progressed little by little and led to the current EU.

However, “sharing of its common inheritance” was not the only goal on the agenda. Since Nation-States were considered to be warmongers, a new way of thinking considered that what is needed is to go beyond traditional allegiance to Nation-States, at the benefit of supra-national structures (such as an increasingly federalist and expanding EU) or infra-national ones (such as what was later called “non-governmental organizations,” NGOs). Furthermore, Europe started to look critically at other aspects of its history (its colonial enterprises, the status of minorities, etc.). What progressively developed was a new ideology which can be termed “postmodernism” (this term is useful since it highlights a shift from “modernism,” as explained below). It is based on the assumption that questioning the inheritance (rather than sharing it) is necessary to avoid the tragedies of the past. This view gained a strong moral influence, especially in conjunction with social and political upheavals in the Western world from the 1960s onwards. It also found philosophical support: postmodern philosophy considers that Western intellectual and cultural values (the heritage of the Enlightenment) have to be “deconstructed” (for more details see footnote 2 and also the Kuntz, 2020).^2^

To avoid all morally reprehensible events, values such as *Democracy, Rule of Law, Human Rights*, etc. are now considered as “Big Principles.” These are of course pre-existing Enlightenment values (meant for emancipation from tyrant powers), but the postmodern thought now considers them as central to the rule of Europe, in a different perspective: “The main goal of the European Union is to defend these values in Europe and promote peace and the wellbeing of the citizens”.^3^ In addition, these Principles have to be exported to the rest of the World (concerning these values and the European will to export them, see relevant texts in Supplementary Material).

There is another important shift to be taken into account. It can be illustrated by, for example, the fact that Churchill spoke about an “act of oblivion against all the crimes and follies of the past” (Churchill, 1946). However, here also, this is not what actually occurred. Instead of oblivion, what developed can be called the “Western Guilt” (Bruckner, 2010). It slowly but surely permeated the values and powers that rule the EU, and to quote the French philosopher Pascal Bruckner: “Having scaled unprecedented peaks of barbarity, the Europe of Brussels has decided to redeem itself by privileging moral values over realpolitik. […] It [modern Europe] has convinced itself that, since all the evils of the twentieth century arose from its feverish bellicosity, it’s about time it redeemed itself and sought something like a reawakened sense of the sacred in its guilty conscience” (Bruckner, 2019).

**FIGURE 1:**
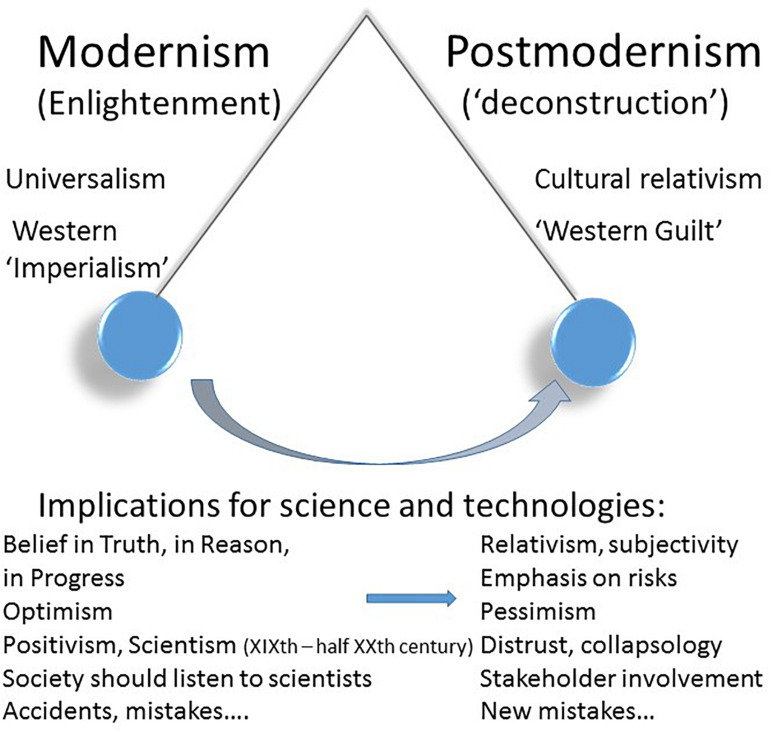
The image of a pendulum to reflect the transition from the modern era to the postmodern era.

In brief: the will of well-doing went too far from the late 1960s onwards. It can be analyzed by the swing from a modernist era (imbedded in the Enlightenment values) to a postmodern one (characterized by the “deconstruction” of these values and by “Big Principles” and guilt). The pendulum (see Figure 1, top part) swung from universalism to cultural relativism, from Western imperialism to Western Guilt. To avoid past mistakes, we are at risk of making new ones, if blinded by Europe’s dream of “no tragedy,” wonderfully sung by John Lennon (*Imagine*):

Imagine there’s no countries

It isn’t hard to do

Nothing to kill or die for

And no religion, too

Imagine all the people

Living life in peace…

However, a great song does not necessarily make a great policy… To take a philosophical reference, Europe has tried to realize Immanuel Kant’s perpetual peace project (Kant, 1795), forgetting however that the idea of peace is an ideal toward which we must strive, but that cannot be simply imposed by “Big Principles.”

This broader historical perspective having been contemplated, it is now possible to analyze the way this postmodern ideology was transposed to science and technologies.

### Postmodernism Transposed to Science and Technologies

Although science and technologies have made a huge contribution to mankind, accidents (such as the Union Carbide disaster in Bhopal), careless use of chemicals (such as pesticides), failure in risk assessments (such as the thalidomide case) and morally reprehensible events also occurred. Many consider that the atomic bombing of Hiroshima and Nagasaki has led to a major change in the way we look at science and has fueled critical views of technology in the postmodern era. The dawn of this era can be set in 1962, when Rachel Carson published her anti-pesticide book *Silent Spring*, although the same decade was still characterized by a peak of admiration of technology during NASA’s Apollo program.

The above-mentioned European dream of “no tragedy” has translated here into a dream of “no technological risk” [see, for example, New Technologies and Eu Law (2017) to illustrate the high prevalence of the theme of “risks” in EU law]. This translation is actually easy to understand if one considers the well-known fact that risk perception is shaped by psychological, social and cultural factors (see Renn and Benighaus, 2013), and even more so if one admits that these factors are rooted in recent History as it happens in Europe.

This new reflection on technologies also found support from sociologists and philosophers [see Kuntz (2012) for a brief history of the growing importance of postmodern thinkers and especially “Science and Technology Studies”]. Therefore, this article will focus on the risk issue in order to illustrate postmodernism influence on science. Other postmodern sociological “studies” dealing with discrimination, such as cultural, gender, intersectionality, etc., are not within the scope of this article.

Here also, to avoid repetition of deleterious events caused by technology, more “Big Principles” were invented: the Precautionary Principle in Europe (see Supplementary Material) and participation of “civil society” in the whole Western world. The latter actually contains two postmodern concepts in a single principle. One is the re-invention of “civil society” (Powell, 2013; Ehrenberg, 2017), also called “stakeholders.” Cicero already spoke about a “societas civilis,” but here it is a different concept, developed from the 1980s: i.e., the rising importance of a supposed *direct* (participative) democracy, as opposed to *representative* democracy especially at the level of Nation-States, which as mentioned above, were considered as potential warmongers or at the service of the industrial and financial oligarchy. In the postmodern sense, direct democracy is not limited to local democracy (the latter is often useful). Transposed to science, it gave rise to the concept of the “democratization of science.” In conjunction with the second concept, “participation,” it has profound implications for science, since it means that scientific processes (such as risk assessment) cannot rely solely on experts, but will benefit from the involvement of “stakeholders” (Kuntz, 2016). Collaboration between medics and patients is often put forward as a successful example of “participatory science.” Although it may be true, this represents a case where no political forces are at work and where all parties want “more science” rather than one party promoting “another science” [see discussion in Kuntz (2012, 2016)].

The report in 2016 by the National Academies of Sciences (United States) entitled “Gene Drive on the Horizon,” although scientifically excellent, illustrates such an ideological shift (led by postmodern sociologists and ethicists on the Committee): it is no longer the society, in its own interest, which should listen to science, but science which should align with “public values” (Kuntz, 2016). Significantly, the subtitle of this report is “Advancing Science, Navigating Uncertainty, and Aligning Research with Public Values.” The term “research” is ambiguous here. It can mean research funding, which is a legitimate political choice and of course will be influenced by “values” (but the ambiguity of the latter term has still to be recognized). It can also be understood as the way science is performed (i.e., the scientific method) and this is problematic: “public values” drastically change according to civilizations and even over time in a given location, which is incompatible with the universalist scientific method.

### New “Big Principles” Also Going Too Far

Regarding the concept of “democratization of science” concept, the following observations should be mentioned. As the GMO dispute has shown, some consider that scientists cannot be trusted [see, for example, attempts to undermine the credibility of EFSA, including at the European Parliament, in Kuntz (2012)]. Such views—that scientific research and risk assessment have to be subject to a democratic process—are encouraged by postmodern theorists who claim that scientific research, as such, cannot be trusted because as a social endeavor it is political by nature and therefore is to be seen as carrying some political untold agenda. This is explicit in postmodern literature and to cite Sheila Jasanoff: “if science is to fulfil its promise of global problem-solving, then there is no other course than to repoliticize it […] by opening up science’s hidden normative presumption to authentic public debate” [Jasanoff (2013); see also other articles in this book].

These views ignore that science is not a matter of democracy, and that this “democracy” is at risk of being captured by the most organized political activists. Of course, scientists can participate in the democratic debate by explaining to a larger public what they know and what they do not know. Here also, these new “Big Principles” went too far: the Precautionary Principle (see Supplementary Material) or, rather, its misuses (Tagliabue, 2016b), encouraged non-science-based regulations and even bans, such as those on GMOs; the participation of “civil society” led to a “soft power” of NGOs. The latter concept has been discussed by many authors and can concern many issues and have positive effects [e.g., diplomacy, human rights, promoting responsible business practice and of course environmental matters; see Katsuji and Kaori (2008); Chambers (2012)]. However, it can also have negative effects such as African governments importing dysfunctional biosafety regulations under the influence of European NGOs amongst others [see Paarlberg (2009) and also below the Golden Rice case]. In addition, in contradiction with the proclaimed goals, these NGOs have no democratic legitimacy.

Here also, the pendulum image (see Figure 1, lower part) illustrates the swing from the Modernism of the Enlightenment, characterized by (maybe excessive) belief in Truth, in Reason, in Progress, optimism about science and technologies, and admittedly its excesses (such as scientism), to Postmodernism, with its cognitive relativism, pessimism about technologies and, consequently, risk evaluation and management becoming ideological and political. An example of the latter shift is illustrated by claims such as: “It is not up to those who fear the occurrence of serious damage to demonstrate plausible grounds for this fear [but up to] those whose actions give rise to the fear of serious damage occurring to demonstrate plausibly why such damage is extremely improbable or scientifically absurd” (Rippe and Willemsen, 2018).

Here also, since mistakes were made during the past, and by trying to avoid their repetition, new ones are made. Europe has managed to export its technological fears and related norms to other countries. This often has negative consequences for these countries, such as depriving them of useful biotechnological tools. Golden Rice is emblematic of such a political outcome: “We Pioneered a Technology to Save Millions of Poor Children, But a Worldwide Smear Campaign Has Blocked It” as recently summarized by Adrian Dubock, Ingo Potrykus, and Peter Beyer.^4^ It is notable that Europe has given much power to the NGOs (partly funded by European sources) that launched this campaign. As Ed Regis also puts it: “every aspect of Golden Rice development, from lab work to field trials to screening, became entangled in a Byzantine web of rules, guidelines, requirements, restrictions, and prohibitions” (Regis, 2019). It is obvious that Europe is at the origin of these regulations.

### Scientists Accepting a New Moral Dogma

There are, of course, differences in the way “Western Guilt” is expressed in the various European States, or in the United States. Obviously, the German *Vergangenheitsbewältigung* (coping with the past) is centered on World War II, while the French *Devoir de Mémoire* (duty of memory) originally derived from the deportation of Jews in France during the Nazi occupation with the aid of metropolitan French authorities, may nowadays be more centered on its colonial past. This shows that a guilty conscience can easily spread from one topic to another (and hence also to science). “Western Guilt” can also take various forms of expression: repentance, seeking forgiveness, shame (which is worse than guilt, an internal sense of moral obligation regarding past faults, while shame is externally generated by the look of others), up to self-hatred. In some countries, this expression may be limited to the way they cope with minorities’ status, or it may take a new form, such as those linked to environmental or food choice issues.

As human beings, scientists from the Western world are also influenced by the mainstream postmodern guilty conscience. This is understandable. What is unfortunate is that scientists often do not react to the ideology that views these tragedies as consequences of the Enlightenment and its “imperialistic” thought, as Postmodernism does. Should scientists display their repentance over historical events that they are not personally responsible for? Isn’t it more useful that scientists perform their missions in society without expiating other peoples’ sins or even developing a self-hatred, obsessed with the errors of the past or with the supposed scientists’ ties with industry for example? A major difficulty is that, in addition to the above-mentioned “Big Principles,” this new moral dogma is often embedded in positive values, such as information, education, research, or an imperative of being “transparent” and “responsible.” It should be noted that, beyond the risk issue, postmodernism can also have a broader influence on scientific activity, imposing new moral obligations on scientists, including for basic research (see my critical view on the imposition of the concept of “Responsible Research and Innovation” to European scientists (Kuntz, 2017)).

### Postmodern Doctrine and Political Actions on Risks

Having depicted the postmodern ideological context, it is possible to examine how it influences the actions of politicians concerning risk in general and more precisely relating to biotechnologies (other risk topics, such as those linked to pesticides, nanotechnologies, etc., are also concerned, but will not be analyzed here).

First, as discussed above, one can observe that postmodernism has a different view on democracy then “modernism.” I propose to call it “democratism” to highlight the shift toward a kind of misplaced democracy and relativism as far as science is concerned. It should be mentioned that the term “democratism” has several quite different meanings in the literature. For some authors, it can simply mean the theory, or principles of democracy. For other authors, it can mean a critic of supposedly “excessive democracy,” which can have several grounds (which will not detailed here), including democracy becoming a form of creed. Obviously, I am using the term more in relation to the second meaning, although not to imply that democracy has been fully replaced by democratism.

The implications for science are: adversarial debates over scientific processes (often promoted by postmodern sociologists as well as by political “Green” or “ecologist” parties and ideologically related organizations), generalization of stakeholder involvement in scientific risk assessment performed by official agencies, and proliferation of various forms of activist science and expertise [see Kuntz (2012, 2016) for examples]. The latter are often welcomed by the media and social media, but have been deplored as “a license to scaremonger” (Grimes, 2019). It should be pointed out that scientists presenting alternative theories against established facts is not a new phenomenon: the French historian of science Alexandre Moatti has compiled many examples, some dating back to the XIX century, and called it “alterscience” (Moatti, 2013). What is new is that this phenomenon has now ideological roots in the sense that postmodernism has made “alterscience” a legitimate expression of “pluralism.” Furthermore, these falsehoods will often appear credible in the postmodern context of distrust in technologies and the “deconstruction” of “normal” science [now considered as just an opinion like any other opinion; to take again EFSA as an example, see how EFSA scientists were confronted with activists at the European Parliament in Kuntz (2012)].

As the GMO case has shown in many countries, this context has favored the radicalization of activists, rather than the opposite, and has contributed to the dilution of established scientific facts. Both have negatively influenced political actions on GMOs. The famous Séralini affair has illustrated how an activist “science” has attracted huge media attention and political over-reaction (Kuntz, 2019).

Postmodernism has also contributed to transforming another pillar of Modernism, namely Judicial Independence, into an increasing “Government of Judges” or “Government by Judiciary” (i.e., a shift in power from politicians to judges). This became possible since, to avoid past abuse of power by dictatorial governments, it was considered necessary to reinforce the concept of Rule of Law (or State of Law), that is to increase the Judicial Discretion concept into a preeminence of, for example, High Courts of Justice over governments (i.e., over democratically elected ones, since authoritarian governments will impose a complete subjection of judges anyway…). The judge, usually ignorant of scientific complexities, will listen to experts from all sides, judging their expressions equivalently and will rule accordingly through “Big Principles” such as the Precautionary Principle.

The objective of this article is not to discuss whether such a shift in power has more positive or negative aspects, but to stress that it is an ideological choice and that it has had an impact on the fate of biotechnology in Europe. The above-mentioned judgment of the CJEU that organisms obtained by mutagenesis (implicitly including gene editing products) are GMOs illustrates this point. This does not imply that this judgment misinterpreted the European GMO Directives: it is fully in the spirit of the European hyper-precautionary ideology which inspired these Directives. In the continuity of this reasoning, there is another example: following the CJEU, and even amplifying its judgment, the French supreme administrative court (*Conseil d’Etat*) in February 2020 gave the government 6 months to adapt the current Environmental Law and 9 months to identify which plants in the catalog of existing varieties have been produced by mutagenesis procedures which appeared after the date of adoption of the 2001 Directive.^5^ This could lead some currently grown plant varieties to be withdrawn from the market. Since this is not at all what the French government wished to do, but will be obliged to do, it illustrates the concept of “Government by Judges”…. In other words, by enacting the first GMO Directive in 1990, European Member States initiated a process they no longer control, partly because of this “Government of Judges.” To be balanced, it should be mentioned that other judicial decisions were more favorable to biotechnology: the *Conseil d’Etat* in 2011 and in 2013 declared illegal the prohibition of GMO cultivation by the French government which had fabricated “scientific” justifications to support its politically motivated ban (Kuntz, 2014) (but a law voted by the Parliament in 2014 banned GM maize cultivation in the country).

Another noteworthy example of the “Government of Judges” is the Dutch Supreme Court’s Climate Judgment that the Dutch state should reduce emissions of CO_2_ from its territory by at least 25% by the end of 2020, as requested by a NGO (see L. Bergkamp’s analysis^6^). Such a ruling may have consequences on the way governments may be considered responsible for omissions in other future court cases [possibly linked to the COVID-19 crisis; see Bergkamp (2020)].

In summary, regarding the issue of technological risks, the consequences of both postmodern democratism and the weakening of elected governments means that politicians will decide in confusion (often catering to NGO lobbying or what they think is the general opinion of their citizens through polls) or may simply obey judges.

### What Differentiates Europe From the United States?

Postmodernism is also rampant in the United States and is expressed for example as “political-correctness,” which has even been described as “The Closing of the American Mind” by the philosopher Allan Bloom (1987). The power of judges also exists. Fears about GMOs have also been propagated by activists in the United States, eventually leading to Public Law 114–216 on GMO labeling, but only in 2016 (i.e., 15 years later than in the EU) and with only minimal labeling requirements. In addition, the Federal government established a formal biotech policy in 1986, the “Coordinated Framework for Regulation of Biotechnology,” which has been since updated. However, it remains a set of principles based on existing laws, not a law in itself. Although the regulations enacted under the Coordinated Framework have limited the deployment of biotech crops to some extent, particularly disease-resistant crops, obviously the United States has not enacted inhibiting laws as did the EU.

So why did we observe a “closing of the mind” in relation to plant biotechnology by European political authorities and not by those in the United States? One of the downstream explanatory factors is that the US regulatory system favors the use of expertise, not popular opinion. In other words, its postmodernism differs from the European one. Obviously, the US authorities consider their national interest and hence those of their industries.

To further understand this, one should look upstream, namely at the broader historical picture. The United States became dominant in North America during the XIX century, and then during the next century it gradually became the “most powerful country on earth”.^7^ Interestingly, this web article explains how the United States chose to become “a European-style imperial power.”

As a comparison, European countries lost this ambition and the EU was not created on might (actually as explained above, it was consciously aimed at basing its policies on values). Actually, the EU itself has none of the classical markers of power (army, permanent seat at the UN Security Council, etc.), not even symbolic ones, which were always inseparable from might since the Ancient World. “Signs and symbols rule the world, not words nor laws,” as Confucius said. Since might appears to be the universal ambition of large political entities since the Ancient World, it is even more striking that Europe has lost such an ambition.

Thus, this indicates that European postmodernism is an unusual development, a reversal, not simply a late phase of modernism. In addition, the EU is not a nation but a conglomerate of diverse nations whose interests may fundamentally contradict each other (as is often observed in many cases).

Keeping in mind that both World Wars devastated Europe, but not the United States, we find a likely historical explanation for the fact that the above-mentioned dream of “no tragedy” is a European not an American one.

The EU has thus given the absolute priority to consumers and perceived environmental care, based on good intentions, but to do so it has indulged itself in excessive regulations for ideological reasons. Europe’s ambition is limited as a soft power, a normative one on environmental, digital, social issues, etc. Due to the size of its single market of more than 500 million inhabitants, Europe has been able to export its standards beyond its borders, not always for the best outcome, as the biotech issue has shown.

## Conclusion and Perspectives

For decades, using rational arguments, scientists tried to convince European politicians of the importance of biotechnology including, more recently, gene editing. Despite the fact that many observers and even politicians are aware that Europe is trailing far behind the United States and now also behind China on plant biotechnology, the trend cannot easily be reverted. Europe’s position is enshrined in an ideology that, like all ideologies, draws an outside line between good and evil: decked out with its “Big Principles,” Europe is convinced it is on the side of great virtue.

In this context, it is difficult to change this ideology, and it was illusory to hope that gene editing products would not be considered as GMOs. Furthermore, it is unlikely that the EU will react appropriately to the risk of becoming a vassal of China and the United States on these new biotechnologies (Martin-Laffon et al., 2019). Unless EU scientists can invoke other “Big Principles” of superior virtue…

Interestingly, the reliance on scientists (virologists, epidemiologists and other specialists) to steer the COVID-19 pandemic marks the return of scientific reality and knowledge with respect to postmodern constructivism, cognitive relativism and stakeholder engagement, *etc*. However, it is premature to conclude from this observation that the postmodern ideology will decline.

## Author Contributions

The author confirms being the sole contributor of this work and has approved it for publication.

## Conflict of Interest

The author declares that the research was conducted in the absence of any commercial or financial relationships that could be construed as a potential conflict of interest.

## Footnotes

1 https://curia.europa.eu/jcms/upload/docs/application/pdf/2018-07/cp180111en.pdf

2 https://www.britannica.com/topic/postmodernism-philosophy

3 https://europarlamentti.info/en/values-and-objectives/values/

4 www.goldenrice.org/PDFs/Dubock,%20Potrykus%20&%20Beyer%20Leaps%20 Mag%202019.pdf

5 www.biotech-gm-food.com/kommentare/french-council-of-state-ruling-on-mutagensis-techniques

6 https://www.huntonnickelreportblog.com/2020/02/dutch-supreme-courts-climate-judgement/

7 https://www.vox.com/2015/5/20/8615345/america-global-power-maps
