# Supplementary material for: Technological Risks (GMO, Gene Editing), What Is the Problem With Europe? A Broader Historical Perspective
Source: Front Bioeng Biotechnol. 2020 Nov 9;8:557115. doi: 10.3389/fbioe.2020.557115 (PMC7681002; doi:10.3389/fbioe.2020.557115)
Supplement: Supplementary file 1 [file Data_Sheet_1.pdf]

Supplementary material to: *Kuntz M (2020) Technological Risks (GMO, Gene Editing), What Is the Problem With Europe? A Broader Historical Perspective. Front. Bioeng. Biotechnol. 8:557115. doi: 10.3389/fbioe.2020.557115*

Further Reading

Postmodernism

The Postmodern Assault on Reason: TNI's interview with philosopher Stephen Hicks by Roger Donway
http://www.mannkal.org/downloads/guests/thepostmodernassaultonreason.pdf

G. Andrade. Standing up for science against postmodernism and relativism
Philosophia: International Journal of Philosophy, 2019, 20(2), 197-211
https://ejournals.ph/article.php?id=14096

European 'values'

What the EU does for its citizens
https://europa.eu/european-union/about-eu/what-the-eu-does-for-its-citizens_en

Goals and values of the EU
https://europa.eu/european-union/about-eu/eu-in-brief_en

Vince projet: Guidelines. Welcome to Europe
https://vince.eucen.eu/wp-content/uploads/2018/06/EN_Welcome_to_Europe_CC_BY_NC_SA.pdf

R. Wike, J. Fetterolf, M. Fagan. Europeans credit EU with promoting peace and prosperity, but say Brussels is out of touch with its citizens
"*In the decades following the devastation and suffering of World War II, the founders of what would become the European Union sought to build a new Europe, "an ever closer union" tied together through economic and political integration, as well as a shared set of values*".
https://www.pewresearch.org/global/2019/03/19/europeans-credit-eu-with-promoting-peace-and-prosperity-but-say-brussels-is-out-of-touch-with-its-citizens/

Europe exporting its 'values'

European Parliament. BRIEFING EU policies – Delivering for citizens. EU support for democracy and peace in the world
https://www.europarl.europa.eu/RegData/etudes/BRIE/2018/628271/EPRS_BRI(2018)628271_EN.pdf

How can Europe promote peace around the world?
https://www.debatingeurope.eu/2019/05/14/how-can-europe-promote-peace-around-the-world/

The European Union's role in providing peace and security: challenges for the new EU team
https://europeanmovement.eu/wp-content/uploads/2014/12/FA-2014-011-Peace-and-Security-ADOPTED.pdf

K. Dervis, J.Mistral. Europe's Crisis, Europe's Future. Brookings Institution, 2014
"*What path, then, should Europe propose ? First Europe can continue to stand for peace, and security through peace… It is now about a "global order" where the objective is to maintain and ensure peace worldwide… through negociations, compromise, and international rules that apply to all equally…*

Europe has been about building a ever larger area of peace. It can be the key player in extending that area to the world as a whole".
https://www.jstor.org/stable/10.7864/j.ctt6wpck8

H. Anastasiou. The EU as a peace building system: deconstructing nationalism in an era of globalization. International Journal of Peace Studies, 2007, 12(2): 31-50
https://www.jstor.org/stable/41852961?seq=1

Precautionary Principle

Science for Environment Policy (2017) The Precautionary Principle: decision making under uncertainty. Future Brief 18. Produced for the European Commission DG Environment by the Science Communication Unit, UWE, Bristol.
https://ec.europa.eu/environment/integration/research/newsalert/pdf/precautionary_principle_decision_making_under_uncertainty_FB18_en.pdf

M. Weimer. Applying Precaution in EU Authorisation of Genetically Modified Products—Challenges and Suggestions for Reform. European Law Journal, 2010, 16(5), 624-657
https://onlinelibrary.wiley.com/doi/full/10.1111/j.1468-0386.2010.00526.x

UK Parliament, Science and Technology Committee. EU regulation on GM Organisms not 'fit for purpose'
https://www.parliament.uk/business/committees/committees-a-z/commons-select/science-and-technology-committee/news/report-gm-precautionary-principle/

S. Rickard. Ploughing the wrong furrow, European Policy Information Center, 2016
http://ieaepicenter1.wpengine.com/publications/ploughing-the-wrong-furrow/

A.L. Stephenson. Germany's Ban of Monsanto's Genetically Modified Maize (MON810): A Violation of International Law. Trade Law & Dev., 2010, 2(2), 292
https://heinonline.org/HOL/LandingPage?handle=hein.journals/traladpt2&div=16&id=&page=

E. R. Mendez, J. R. Lupien. FAO/WHO food standards program: codex alimentarius. In Food Safety Handbook, Ronald H. Schmidt, Gary E. Rodrick, eds. Wiley-Interscience, 2003
"*The WTO considered the US complaint that the EU ban was too restrictive and was not based on sound scientific evidence… The EU, however, has in effect ignored this decision and maintained its ban, invoking amongst other things the "precautionary principle", consumer demands and other non-science-based factors*".
https://gavsispanel.gelisim.edu.tr/Document/bgcol/20190220114437735_b416cd2a-7723-40f6-8392-7d12b21b66de.pdf

L. Bergkamp, J.C. Hankamp. European Food Law and the Precautionary Principle: Paradoxical Effects of the EU's Precautionary Food Policies: A Legal-Economic Perspective. In Regulating and Managing Food Safety in the EU, Springer, 2008, Pages 217-244
https://link.springer.com/book/10.1007/978-3-319-77045-1
